# Systemic inflammatory response syndrome (SIRS) induced by carbon tetrachloride in rats

**DOI:** 10.1080/09629359791974

**Published:** 1997-02

**Authors:** J. Gergely, S. Sipka, J. Csípő, M. Udvardy, Gy. Szegedi, A. Kulcsár

**Affiliations:** 1 Department of Pharmacology University Medical School Debrecen Debrecen H-4012 Hungary; 2 III. Medical Clinic University Medical School Debrecen Debrecen H-4012 Hungary; 3 II. Medical Clinic University Medical School Debrecen Debrecen H-4012 Hungary

## Abstract

We have observed the symptoms of systemic inflammatory response syndrome (SIRS) in male rats intoxicated by carbon tetrachloride (CCl_4_). Severe hypothermia, tachypnoea and increase in the heart beat min were diagnosed. These symptoms developed in the first hour of intoxication. The hepatic dysfunction was characterized by elevated bilirubin levels. In the sera we have measured increases in the activity of secretable (group II) phospholipase A_2_ sPLA_2_ (2,8x) and 6-ketoprostaglandin F_1α_ (KPGF) (1,44x). Supposedly the free radicals derived from CCl_4_—mainly trichloromethyl—could induce the prompt reaction of SIRS and the release of sPLA_2_ as well as the formation of KPGF. Our findings show that in the early phase of CCl_4_ intoxication the symptoms of SIRS can be related to elevation of sPLA_2_ and the products of cyclooxygenase II.


Mediators of Inflammation, 6, 73–74 (1997)

### Systemic inflammatory response syndrome (SIRS) induced by carbon tetrachloride in rats

J. Gergely,[1,CA], S. Sipka,[2], J. Csípő, [2], M. Udvardy,[3] Gy. Szegedi[2] and A. Kulcsár[3]

[1]Department of Pharmacology, [2]III. Medical Clinic, [3]II. Medical Clinic, University Medical School, Debrecen, H-4012 Debrecen, Hungary

[CA]Corresponding Author
Fax: (+36) 52 316743

**We have observed the symptoms of systemic inflammatory response syndrome (SIRS) in male rats intoxicated by carbon tetrachloride ($CCl_4$). Severe hypothermia, tachypnoea and increase in the heart beat/min were diagnosed. These symptoms developed in the first hour of intoxication. The hepatic dysfunction was characterized by elevated bilirubin levels. In the sera we have measured increases in the activity of secretable (group II) phospholipase $A_2$ $sPLA_2$ (2,8x) and 6-ketoprostaglandin $F_{1\alpha}$ (KPGF) (1,44x). Supposedly the free radicals derived from $CCl_4$—mainly trichloromethyl—could induce the prompt reaction of SIRS and the release of $sPLA_2$ as well as the formation of KPGF. Our findings show that in the early phase of $CCl_4$ intoxication the symptoms of SIRS can be related to elevation of $sPLA_2$ and the products of cyclooxygenase II.**

**Key words:** Acute $CCl_4$ intoxication, SIRS, $sPLA_2$, 6-ketoprostaglandin $F_{1\alpha}$

### Introduction

Actual response of the living organism to various injuries depends not only on the type of the noxa but also on the reactivity of the body. Based on several observations, a new concept, the systemic inflammatory response syndrome (SIRS) was introduced in 1992.[1] SIRS was defined as an acute response to different forms of stress, e.g. infection, tissue necrosis, combustion etc. The characteristics of SIRS were determined by the existence of (1) hypothermia or hyperpyrexia, (2) high pulse rate, (3) increased respiratory frequency, (4) leukocytosis or leukopenia. If two of these signs have been developed the syndrome is manifested. Carbon tetrachloride ($CCl_4$) is the most potent halogenated hydrocarbon. In the hepatic microsomes it is activated to free radicals, mainly trichloromethyl ($CCl_4$–$CCl_3$) bound covalently to proteins, nucleic acids or lipids. Free radicals can mediate numerous toxic effects including membrane damage, diffuse fatty degeneration and necrosis of the hepatocytes in zone III of the liver acinus.[2] Some consequence of the acute $CCl_4$ intoxication can develop within a few hours.[3] The liver is deeply involved in the processes. Hepatocytes are stimulated by pro-inflammatory cytokines, e.g. $TNF_\alpha$. At the same time they also secrete acute phase proteins.[4] The elevation of serum bilirubin is an early marker of acute toxic hepatic failure.[5] Different non-specific stimuli can induce the release of secretable (group II) phospholipase-$A_2$ ($sPLA_2$), initiating the production of arachidonic acid and a cascade of enzymatic reactions, e.g. cyclooxygenase mediated prostaglandin synthesis.

### Experiments

Male Wistar rats (200–220 g bodyweight) were treated with a single $CCl_4$ dose of 1.25 ml/kg between 08.00 and 09.00 h. Controls received saline in the same volume and time. In the first hour of intoxication body temperature decreased from 36.3°C to 31.6°C. A tachypnoea was registered, respiratory frequency augmented from 68 to 110/min and heart beat/min increased from 341 to 368. The rise in serum bilirubin levels reflected liver lesions in the animals. Furthermore elevated activities of $sPLA_2$ were found in sera (2,8x) by using: 1-stearoyl-2-(1-$^{14}$C)arachidonyl,L-3-phosphatidylcholine (Amersham) as substrate, and increased amounts of 6-ketoprostaglandin $F_{1\alpha}$ (KPGF) production (1,4x) were measured in aortic tissue specimens.[6]

Results are given in Table 1.

### Discussion

In our experiments three symptoms of SIRS, hypothermia, tachypnoea and increased pulse rate were observed in the 60 min of acute $CCl_4$ intoxication of rats. Vadas and Pruzansky demonstrated that secretory non-pancreatic

© 1997 Rapid Science Publishers

**Table 1.** Changes induced by $CCl_4$ in rats (mean ± SE)

| Experimental animals | Controls saline (n = 15) | $CCl_4$ (60 min) (n = 10) |
|---|---|---|
| Body temperature °C | 36.3 ± 0.7 | 31.6 ± 0.3 (P < 0.001) |
| Respiratory frequency/min | 68 ± 3.5 | 110 ± 5 (P < 0.001) |
| Heart beat/min | 341 ± 10.4 | 368 ± 8.7 (NS) |
| Serum $PLA_2$ U/l | 0.001 ± 0.0012 | 0.0028 ± 0.0035 (P < 0.001) |
| 6-keto-$PGF_{1\alpha}$ in aortic tissue pg/mg protein | 182 ± 41.0 | 263 ± 89.7 (P < 0.001) |
| Serum bilirubin μmol/l | 3.13 ± 0.13 | 3.84 ± 0.42 (NS) |

*P vs controls.*

$PLA_2$ correlated with markers of multi system organ injury and SIRS.[7] We also found elevated $sPLA_2$ activities serving a further support to our suggestion that acute $CCl_4$ intoxication is also a form of SIRS. The products of $sPLA_2$, platelet activating factor (PAF) and arachidonic acid, the source of prostanoids may have secondary but very important mediating role in the completion of the multiple organ failures. The increased levels of KPGF reflected an increased prostanoid synthesis by cyclooxygenase II.[8–10] Liver lesion, hypothermia, tachypnoea and tachycardia manifested together represent a real form of multiorgan failure. The conclusion of our observation is that during acute $CCl_4$ intoxication a form of SIRS is manifest.

### References

1. ACCP/SCCM. Consensus Conference: definitions for sepsis and organ failure and guidelines for the use of innovative therapies in sepsis. *Crit Care Med* 1992; **20**: 864–869.
2. Rappaport AM. Toxic injury of the liver. In: Farber E, Fischer M, eds. *Physio anatomical Basis of Toxic Liver Injury.* New York, Basel: Marcel Decker, 1980.
3. Kalf GF, Post GB, Snyder R. Solvent toxicology. *Ann Rev Pharm Toxicol* 1987; **27**: 399–427.
4. Falus A. Cytokinek és a máj. In: *Liver and Immunosystem*, conference proceedings, Pécs, 1994.
5. Fogarasi M, Kubala É, Bíró J, Falus A, Nemesánszky E. Interleukin-6: Értünk vagy ellenünk? Újabb eredmények az IL-6 klinikai vonatkozásairól. *Orvosi Hetilap* 1994; **135**: 2075–2082.
6. Inoguchi T, Umeda F, Ono, H, Kunisaki M, Watanabe J, Nawata H. Regional myocardial functional and electrophysiological alterations after brief coronary artery occlusion in conscious dogs. *J Clin Invest* 1975; **56**: 978–983.
7. Vadas P, Pruzansky W. Cytokine-induced phospholipase $A_2$ expression in the systemic inflammatory response syndrome. In: *3rd International Conference on Lipid Mediators in Health and Disease*, Jerusalem, 1993, A 47.
8. Meade EA, Smith WL, DeWitt DL. Differential inhibition of prostaglandin endoperoxide synthetase (cyclooxygenase) isozymes by aspirin and other non-steroid antiinflammatory drugs. *J Biol Chem* 1993; **344**: 4610–4614.
9. Tordjman C, Coge F, Andre N, Tordjman C, Coge F, Andre N, Rigue H, Spedding M, Bounet J. Characterization of cyclooxygenase 1 and 2 expression in mouse resident peritoneal macrophages in vitro. *Biochem Biophys Acta* 1995; **1256**: 249–256.
10. Pritchard KA Jr, O'Banion MK, Miano JM, Vlasic N, Bhatia VG, Young DA, Stemerman MB. Induction of cyclooxygenase-2 in rat vascular smooth muscle cells in vitro and in vivo. *J Biol Chem* 1994; 269: 8504–8509.

ACKNOWLEDGEMENT. This work was supported by a grant from the Ministry of Public Welfare and the Health Scientific Committee (T 02 425/93).

**Received 27 October 1996; accepted 28 October 1996**
